# Integration gaps persist despite immigrants’ value assimilation: evidence from the European Social Survey

**DOI:** 10.3389/fsoc.2025.1504127

**Published:** 2025-04-01

**Authors:** Jorge Suárez, Ivar R. Hannikainen

**Affiliations:** Department of Philosophy I, University of Granada, Granada, Spain

**Keywords:** migration, multiculturalism, civic integration, assimilation, ingroup bias

## Abstract

In Europe, although integration of the immigrant population is acknowledged as a multidimensional challenge, the precise dimensions of integration have varied considerably throughout the past decades and between nations. Nowadays, most states have adopted ‘civic integration’ programmes to some extent, thereby placing weight on the acquisition of ‘national moral values’, implying that successful integration requires the assimilation of certain core values and implementing various strategies to instil these in immigrants. However, critics of civic integration have called into question whether the adoption of a host society’s normative values facilitates immigrants’ own integration. To provide evidence on this matter, we leverage data from the European Social Survey, collected between 2002 and 2020 (*N* = 261,830) and examine how immigrants’ self-reported values relate to their integration. Our analyses ask whether value assimilation predicts improvements in immigrants’ occupational status, socialization, and political participation throughout 27 countries in the European Union. We find that differences in moral values account, at most, for a fraction of the integration gap between natives and immigrants. These results therefore call into question the assimilationist principle that adopting a host society’s values is conducive to immigrants’ integration.

## Highlights

- Relative to natives, immigrants in Europe face disparities in the workplace, in their social integration and in their political participation.
- Immigrants assimilate over time, approximating normative levels of human values in their native reference group.
- In general, calibration of human values to the host society only weakly predicts integration.
- Elevated endorsement of conservation values may somewhat hinder immigrants’ political participation.

## Introduction

Born in the Netherlands, Nancy Holten moved to Switzerland when she was a child. By 2017, she had lived in Swiss territory for thirty years, gained fluency in Swiss German, and raised two children. Though otherwise a cherished member of her community, Nancy was also an activist for animal rights, who openly denounced certain Swiss traditions, such as hunting, piglet racing, or cow bells, as part of her animal rights advocacy. In the Swiss system, where naturalisations are approved initially by the administration, and then voted on by national residents living in the applicant’s municipality, this resulted in Ms. Holten’s citizenship application being twice denied on the grounds that she had not integrated into the Swiss way of life.

This case is a particularly visible example of a recurring phenomenon throughout multiple European countries: immigrants’ successful naturalization often depends not only on their demonstrable integration—e.g., through education, employment, and linguistic fluency—but also on their allegiance to the host society’s norms and values. At the root of this phenomenon lies a difficult sociopolitical question that liberal democracies have faced with each new wave of immigration throughout the last century (Dustmann and Frattini, 2013): To what extent, and in which ways, must newcomers adapt to their receptor communities?

State-level guidelines have generally focused on the fulfilment of social and material needs for the immigrant population, such as having a fair income, being socially integrated into their local communities, and actively participating in politics or civic organizations (Netherlands Institute for Social Research, 2012). Yet recently many European states have shifted toward treating civic integration as an increasingly relevant aspect of immigrants’ integration process. Critics, however, argue that these policies may not be compatible with liberal democratic ideals, especially when they are focused on scrutinising the person’s inner dispositions and values, or their degree of value assimilation (Joppke, 2017; Joppke and Bauböck, 2010). Since the justification for these measures rests on the assumption that civic integration is part of the full integration processes of immigrants, it is important to understand whether civic integration facilitates immigrants’ broader integration process.

In the present work, we provide fodder for this debate by examining the associations between value assimilation and immigrants’ integration processes in the domains of political participation, social life, and occupational status, across several European countries. Does the assimilation of host societal values by the immigrant population relate to these other dimensions of their integration? As a contribution to the current research topic, “The Citizenship of International Migrants: Rethinking the Migration-Citizenship Nexus Today,” this transnational empirical study aims to shed light on the recent debates about civic integration and its place in liberal democracies’ understandings of citizenship. In addition to this, our findings may also help to reconsider the legitimacy and justification of migration policies currently in place throughout many European states.

### The shift toward civic integration

When individuals who have socialized in one specific culture face the prospect of moving to a new cultural community, they may resort to different strategies to adapt. Berry (1997) famously distinguished four different processes: assimilation, integration, segregation, and marginalization. The first two strategies, both of which are central to our paper, have in common the adoption of the host society’s culture by the newcomer. They differ, however, in that assimilation implies abandonment of the original culture, whereas integration refers to a reconciliation between original and host cultures.

These strategies are often discussed in connection to how immigrants *adapt*, but the same models have also given rise to distinct migration *policies* which place distinct demands on newcomers. Throughout much of the 20th century, *assimilation* was the predominant approach toward immigrants’ process of adaptation to the host society. Then, in the 1960s, *integration* progressively replaced it in official European guidelines on what policies states should implement for newcomers. It was not until the beginning of the 21st century that integration became the main paradigm in European immigration policy (Perchinig, 2012), after many Western democracies abandoned the multiculturalist approach (Joppke, 2004)—a trend that some scholars have attributed to its broad-based denial of value homophily (McPherson et al., 2001; Rauwolf et al., 2015) as a precursor of social trust. Since then, a fruitful literature has advocated a shift toward the so-called *civic integration* among European democracies (Joppke, 2017; Joppke, 2007). In practice, this has implied that a growing number of countries are implementing policies that require immigrants to prove their adaptation to national values and customs, beyond their mere capacity to speak the primary official language (Perchinig, 2012).

The idea of integration has evolved since Berry’s proposal, and now includes other sociomaterial aspects. In this paper, we understand integration as a holistic approach that goes beyond Berry’s concept of integration and focuses on immigrants’ acquisition of the appropriate resources and capacities in several different facets to prosper in the host community. Although policy advisors and academics now agree that integration is a multidimensional construct, they differ in their precise definition (Penninx and Garcés-Mascareñas, 2016; Spencer and Charsley, 2021), and only recently have efforts to create generalisable indexes gained traction (Harder et al., 2018).

On the institutional side, the EU has produced different documents that aim to identify and shape the integration strategies of its Member States. Although the EU recognises countries’ autonomy to design and implement their own integration policies, the guidelines published by EU bodies implicitly seek and promote harmony between different national models (Joppke, 2007). According to these documents, integration is defined as a “dynamic, two-way process of mutual accommodation by all immigrants and residents,” while also claiming that “integration implies respect for the basic values of the European Union” including “respect for the principles of liberty, democracy, respect for human rights and fundamental freedoms, and the rule of law” (Council of the European Union, 2004). The fact that the Council of the European Union explicitly demands immigrants’ adherence to the fundamental values of the European Union represents a clear departure from the multiculturalist project that had previously prevailed (Larin, 2020; Orgad, 2019), with some authors even espousing a certain return to assimilationist perspectives (Brubaker, 2001). More recently, EU guidelines on integration have explicitly articulated civic integrationist ideals in its Action Plan for Integration and Inclusion. In this Action Plan, one of the key actions is to foster “an understanding of the laws, culture and values of the receiving society as early as possible, for example through civic orientation courses” (European Commission, 2020). This resonates with past assimilationist views, for which “cultural assimilation, or acculturation, is likely to be the first of the types of assimilation to occur when a minority group arrives on the scene” (Gordon, 1964), leaving unanswered the question of whether this process is either a facilitator or a byproduct of integration in other domains.

Thus, civic integration is considered part of a myriad of dimensions which together compose full integration. European states dictate their own integration demands in order to grant residency, citizenship, or even nationality to immigrants. The list varies across countries, ranging from the widely accepted and minimal requirement of compliance with the law to the more controversial expectation that immigrants know and have internalized national customs. Whether civic integration is compatible with liberal democratic ideals has been debated elsewhere (Joppke and Bauböck, 2010). In the present work, we will set aside this question and ask whether civic integration can be shown to predict improvements in immigrants’ lives in other domains—such as their occupational, social, or political integration.

### The role of values in integration

Personal and moral values predict an individual’s performance in numerous areas of life (e.g., (Abdallah, 2023; Acemoglu, 2021; Anglim et al., 2022; Ismail et al., 2019; Kruse et al., 2019)), and are greatly influenced by how they perceive the values of others (Pronin et al., 2004; Zou et al., 2009). This predicts that immigrants’ values might flexibly adapt in order to help them succeed when settling into a new community. Relatedly, Abu-Rayya and colleagues (Abu-Rayya et al., 2023) found that immigrants’ own values predict their motivation to adopt various potential integration strategies. Past research suggests that immigrants naturally undergo integration as they spend time in the host country, developing social networks (Koelet and de Valk, 2016), entering the job market (Bowlus et al., 2016), and availing themselves of social services (Dias et al., 2011; Suárez, 2022), with similar integration rates for most ethnic groups and dimensions (Heath and Schneider, 2021).

At the same time, a large literature has documented the public perception that immigrants pose both economic and cultural threats to the host society. On the economic front, restrictive attitudes toward immigration often stress the economic impact of immigrants’ use of welfare services, such as free healthcare or unemployment subsidies, and their participation in the job market (Carens, 2005; Degen et al., 2019; Miller, 2009; Römer, 2017). On the cultural front, the diversity that waves of immigration bring is often perceived as destabilising the society’s traditions, as well as diluting its identity and uniqueness—an issue that has been critically discussed among proponents of cosmopolitanism (Appiah, 2007), multiculturalism (Banting and Kymlicka, 2009), open borders (Carens, 2013), and closed borders (Miller, 2014), among others.

Experimental research on this question has demonstrated that threats to national identity are considerably more likely to engender exclusionary attitudes than are economic threats (Burgoon, 2014; Sniderman et al., 2004). This literature has emphasized natives’ perceptions (Putnam, 2007; Sortheix and Schwartz, 2017) and attitudes (Albertson and Gadarian, 2012; Brader et al., 2008; Hainmueller and Hopkins, 2014) toward immigrants, while comparatively less work has examined how *immigrants’* values bring to bear on their integration process. One exception is Alejandro Portes and colleagues’ research on segmented assimilation (Portes et al., 2005; Portes and Zhou, 1993), which focuses on how the children of immigrant parents reconcile host and parental cultures and its downstream impact on integration success. This neglect of immigrants’ experience may ultimately lead to a mismatch between what integration policies promote and demand from immigrants and what is ultimately conducive to their successful integration.

To contribute to the debate on whether civic integration policies are related to immigrants’ broader integration, in the present research we first ask (RQ1) whether immigrants’ values differ on average from those of natives in the European context and whether any such differences wane over time or not—providing a test of assimilation. Second, we examine (RQ2) whether and how values relate to occupational, social, and political integration outcomes for the whole population—for natives and immigrants as a whole. Third, we explore (RQ3) whether value assimilation is associated with a reduction in the integration gap between natives and immigrants—a question that brings to bear the current political demand for civic integration among European states.

## Methods

### Data

To address these three research questions, we employ data from the European Social Survey (ESS). The ESS is a cross-national study carried out every two years, since 2002, in approximately 30 European countries. With the number of participants varying from 30,000 to over 50,000 in each round, the ESS collects nationally representative data on values and attitudes, as well as numerous sociodemographic measures. Thus, the present article presents a retrospective study of archived data. All data can be downloaded from the ESS website (www.europeansocialsurvey.org) and in this study it was last accessed and downloaded on the 13^th^ of November 2024. The ESS Research Ethics Board guarantees that all studies abide by the Declaration on Professional Ethics of the International Statistical Institute and that analysts have no access to participants’ identifying information. Prior to participation, informed consent was obtained from respondents.

We accessed rounds 1 to 10 [from 2002 (1st round) to 2020 (10th round)] of the ESS in the subset of 28 countries that belonged to the EU during this period—except Croatia, which joined in 2013 but was still included—with the absence of data from Malta. We included adult participants between the ages of 18 and 65 (both included). Underage participants were excluded due to difficulties in interpreting integration in adolescence stages, and adults over 65 years old were excluded due to peculiarities of this age group: (i) the so-called ‘salmon bias’, according to which the elderly immigrant population is underrepresented and partially selected (Di Napoli et al., 2021; Lu and Qin, 2014), and (ii) the fact that many European countries set the retirement age at around 65 years old, confounding the measurement of occupational integration. This resulted in a final data set (*n* = 261,830) comprising ten waves (*k* = 10) from 27 (*j* = 27) EU countries, although some of these countries did not offer data for all of the waves used, making the final database incomplete in some country-wave combinations. All analyses were carried out in R version 4.3.1 (R Core Team, 2023). It is worth mentioning that Rounds 1 through 9 (2002–2018) consistently collect responses using face-to-face interviews, while Round 10 (2020) changed to a hybrid mode of data collection in several countries where respondents self-administered the questionnaire. Additionally, Round 10 also lacks responses to Schwartz’s values scale in 10 countries. In Supplementary Analysis 1 we report the results of our analyses separately for each wave to justify the decision to merge all waves in a single aggregate analysis.

### Measures

In this study, respondents were coded as natives if their current country of residence matched their country of birth, and as immigrants otherwise. This results in a dichotomous grouping variable that distinguishes autochthonous from foreign-born respondents. Although this coding is analytically useful to capture a large share of immigrants, it obscures different migration backgrounds like second-generations or the so-called ‘decimal generations’ (Oropesa and Landale, 1997), that can be relevant to further analyses.

To examine how immigrants’ integration processes are influenced by their values, we searched for indicators of integration (which we treat as dependent variables), moral values (which we treat as explanatory variables), and sociodemographic information in the ESS database.

### Integration outcomes

#### Occupational integration

Newcomers face difficulties entering the job market, with an initial unemployment gap that tends to reduce as they spend more time in the country and, consequently, in the job search (Ballarino and Panichella, 2015). Although some authors have also explored differences in the occupational status of immigrants and natives (Bowlus et al., 2016), other studies focus on mere participation in the labor market as the key measure of integration in this domain (Fleischmann and Dronkers, 2010). In our analyses, we adopt the latter approach, employing a single-item dichotomous measure [pdwrk] of occupational integration, which asks participants if they have done any paid work in the 7 days before the interview, where 1 = ‘yes’ and 0 = ‘no’.

With this coding, we capture whether native and foreign-born interviewees are an active part of the labor market. However, this way of operationalising occupational integration disregards both personal and structural aspects of this dimension, i.e., whether the person has decided not to or is not able to work—e.g., students, homemakers, or young pensioners—or whether their paid work is stable or commensurate with their qualifications.

#### Social integration

Creating a new social network after arrival in a new country is a challenge. Social integration comprises the objectives of acquiring enough contacts in the host community to cover the usual social needs for well-being, and of establishing social relationships with other native peers—and not only with other immigrants. Goel and Lang (2009) demonstrated that immigrants with more close friends and relatives have better chances of finding a job and are less prone to loneliness (Koelet and de Valk, 2016; ten Kate et al., 2020).

In this paper, we operationalized social integration as the acquisition and maintenance of social bonds—regardless of the native composition of such bonds. The constructed variable is a composite index of two responses: [(sclmeet)] how often [do you] socially meet with friends, relatives, or colleagues; and [(sclact)] how often [do you] take part in social activities compared to others of the same age. Given that the response scales for each question differed, the responses were first standardised (or z-scored), thus resulting in two variables with means of 0 and standard deviations of 1:


$$Z\left(i\right)=\frac{i-M\left(i\right)}{SD\left(i\right)}$$

Next, we averaged the standardized [sclmeet] and [sclact] measures to create the social integration index:


$$\mathrm{Social}\_\mathrm{integration}=\frac{Z\left(\mathrm{sclmeet}\right)+Z\left(\mathrm{sclact}\right)}{2}$$

This measure aims to capture how socially active respondents are in society, although the meaning of the answers could be interpreted differently for natives and foreign-borns in a more detailed analysis, i.e., by analysing not only how socially active they are, but also the actual composition of their social network and the kind of activities carried out by the respondent—and, consequently, the actual impact on their integration process.

#### Political integration

Political integration is considered among the hardest dimensions of integration to improve among immigrants (Chaudhary, 2018; Giugni and Morales, 2011; Morales and Morariu, 2011). We based our measure of political integration on previous research on political participation (Cinalli and Giugni, 2011; Wright and Bloemraad, 2012), creating a composite index of five variables capturing a series of politically relevant behaviors included in the ESS instrument throughout the whole time series: [i, (contplt)] having contacted a politician or government official, [ii, (badge)] having worn or displayed campaign badge/sticker, [iii, (sgnptit)] having signed a petition, [iv, (pbldmn)] having participated in public demonstrations, and [v, (bctprd)] having boycotted certain products. Responses were coded as 0 if the behavior had not been carried out in the 12 months before the interview, and as 1 if it had. The constructed index averaged participants’ responses to all five variables, thus resulting in an index ranging from 0 (no political participation) to 1 (maximum political participation).

#### Human values scale

**Figure 1:**
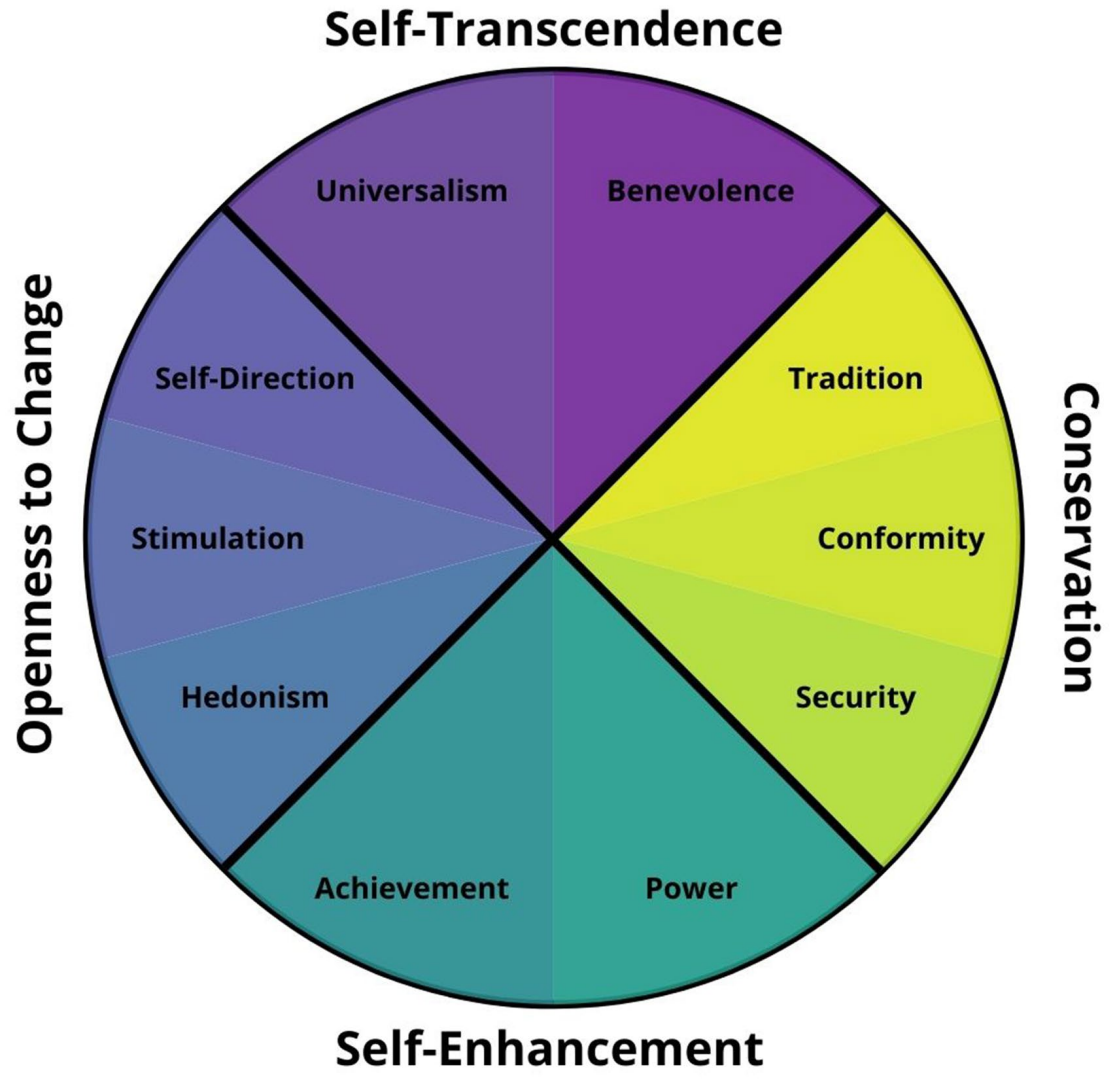
Schwartz’s model of human values. Adapted from Schwartz (1992).

Social psychologist (Schwartz, 1992) created the Human Values Scale, which states that the basic human values found in all modern countries can be divided into at least ten separate categories. Figure 1 illustrates how these values can be organized in a two-dimensional plane. Values associated with the subject’s self-transcendence or self-enhancement are represented along the vertical axis. The bottom displays values that relate to personal achievement and self-improvement, while the top reflects values that relate to ideals of caring for others and entities beyond the self. The horizontal axis contrasts values of openness and conservation. Values on the right represent conservative views that favour stability and order, whereas values on the left represent more dynamic views that favour diversity and change. According to Schwartz’s model, value systems can be described along these four dimensions. Thus, all social communities exhibit these four sets of values, although each one varies in their magnitude and distribution.

The Human Values Scale has been a part of all ten ESS rounds since 2002 and contains 21 items that present a fictional character who identifies with a certain value and ask respondents to rate their level of self-identification. Responses are rated from 1 (“Very much like me”) to 6 (“Not like me at all”) on a Likert scale. The scale was reverse-scored for clarity such that higher values correspond to the maximum self-identification. In the present article, we have grouped the 21 items according to the superstructure of 4 factors, and all four individual difference measures revealed good internal validity: self-transcendence values related to universalist ideals (Cronbach’s *α* = 0.75); self-enhancement values related to one own’s prosperity (*α* = 0.75); conservation values related to preservation and conformity (*α* = 0.73), and openness values related to open-mindedness and personal freedom (*α* = 0.70).

In our study, this scale will serve to operationalize (i) the adherence to each set of value and (ii) the degree of value assimilation among the immigrant population for each of them, by calculating the difference between each individual and their native reference group. As a result, this difference score represents the dissimilarity between a participants’ value profile and the mean response in their reference group, but changes in these difference scores cannot straightforwardly be interpreted either as longitudinal approximation of the individual to their reference group or of the reference group to the individual.

### Sociodemographic variables

We also include the respondent’s age, gender, and educational attainment—which is harmonized between countries in the ESS as a 7-level ordinal variable following ES-ISCED categories—as control variables to mitigate the impact of sociodemographic confounds and identify the independent effect of immigrant versus native backgrounds.

By dividing the years each respondent has lived in the host country by their age, we calculated the proportion of life spent in the host country, which enabled the representation of immigrants’ share of life in the host country—a brief description and justification of this measure can be found in Supplementary Analysis 4. Table 1 provides summary statistics of the measures in this study, separating the subset of natives (*n* = 241,034) and immigrants (*n* = 20,796).

**Table 1:** Descriptive statistics.

		Natives		Immigrants	
		% or mean	*n*	% or mean	*n*
Sociodemographic measures	Age	*42.7*	241,034	*42.6*	20,796
	Proportion of life spent in host country (years residing/age)	100	241,034	48.48	20,796
	Gender: female	53.13	128,024	54.20	11,270
	Education				
	ES-ISCED I, less than lower secondary	5.20	10,533	8.41	1,495
	ES-ISCED II, lower secondary	15.04	30,476	15.65	2,780
	ES-ISCED IIIb, lower tier upper secondary	19.21	38,916	14.07	2,499
	ES-ISCED IIIa, upper tier upper secondary	25.46	51,578	20.00	3,554
	ES-ISCED IV, advanced vocational, sub-degree	11.28	22,862	13.72	2,438
	ES-ISCED V1, lower tertiary education, BA level	11.84	23,983	11.87	2,109
	ES-ISCED V2, higher tertiary education, > = MA level	11.97	24,259	16.27	2,891
Schwartz’s human values	Self-transcendence	*3.84*	241,034	*3.96*	20,796
	Openness	*3.14*	241,034	*3.16*	20,796
	Self-enhancement	*2.66*	241,034	*2.74*	20,796
	Conservation	*3.29*	241,034	*3.43*	20,796
Integration outcomes	Occupation: employed	66.61	241,034	63.82	20,796
	Social life	*0.005*	241,034	*−0.062*	20,796
	Political participation	*0.172*	241,034	*0.154*	20,796
Total *N*		261,830			

Italicized values represent means.

### Analysis plan

The analyses presented here seek to determine whether immigrants’ assimilation of host societal values is positively associated with successful integration in other domains. First, we investigate whether there are group differences (i.e., between immigrants and natives) on the three integration outcomes, and in self-reports of Schwartz’s human values. Drawing on the proportion of life measure, we also ask—as part of Research Question 1—whether any such differences diminish the longer immigrants spend in the host country, as implied by value assimilation. Next, in Research Question 2, we examine the relationship between Schwartz’s human values and integration outcomes, applying conditioning methods to rule out potential confounding effects of the proportion of life in the host country. Our analytic approach to Research Questions 1 and 2 employs logistic (for occupational status) and linear (for social life and political participation) mixed-effects regression models with a random intercept of country of residence. In every statistical model, we enter the respondent’s age, gender, and level of educational attainment—treated as continuous in the models due to its monotonic effects on all dependent variables—as covariates (i.e., fixed effects)—to adjust for differences in integration and human values attributable to these sociodemographic characteristics. We apply analysis weights in the models to account for deviations from the stratified sampling procedure due to nonresponse and sampling error and to account for population differences between countries.

Lastly, to answer Research Question 3, we evaluate whether group differences in human values account for the integration gap between immigrants and natives, by comparing the average direct and indirect effects in parallel mediation models. The mediation models evaluate whether the observed relation between being foreign-born and integration outcomes is better explained via human values, i.e., whether—at least a fraction of—this relation is due to the effects that being foreign-born has on acquiring certain values and, through such values, on integration. In these analyses, we report the indirect effects of foreign-born status on all three measures of integration via each of the four human values, as well as its direct effects, using the *lavaan* package in *R* (Rosseel, 2012).

The three main research questions and a summary of the present findings are reported in Table 2.

**Table 2:** Summary of study findings.

(RQ1) How do immigrants’ values differ from natives’? How does this difference evolve as immigrants spend a larger share of their lives in the host country? Immigrants report greater self-identification with the four sets of Schwartz’s human values (*p*s < 0.026), with greater differences for self-enhancement and conservation. This difference significantly reduces as immigrants spend a larger proportion of their lives in the host country for the two latter sets of values (*p*s < 0.001), but very slightly increases for self-transcendence (*p* = 0.012) and openness values (*p* < 0.001).
(RQ2) Do values relate to occupational, social, and political integration outcomes? Occupational status is negatively related to self-transcendence and conservation, while positively related to self-enhancement and unrelated to openness. Social integration is related positively to self-transcendence and openness, negatively to conservation, and unrelated to self-enhancement. Political integration is strongly positively related to self-transcendence and less to openness, while strongly negatively related to conservation and less to self-enhancement. Deconfounding the analyses by conditioning on proportion of life supports the idea that, for the foreign-born population, values have small although significant relations to integration indicators
(RQ3) Do immigrant-native differences in human values explain disparities in integration outcomes? Parallel mediation models show that values mostly account for a negligible portion of the integration gap, in comparison to the direct effect of being foreign-born. The association between conservation values and political integration constitutes an exception to this: these values account for up to 54% of the total effect on political participation.

## Results

**Figure 2:**
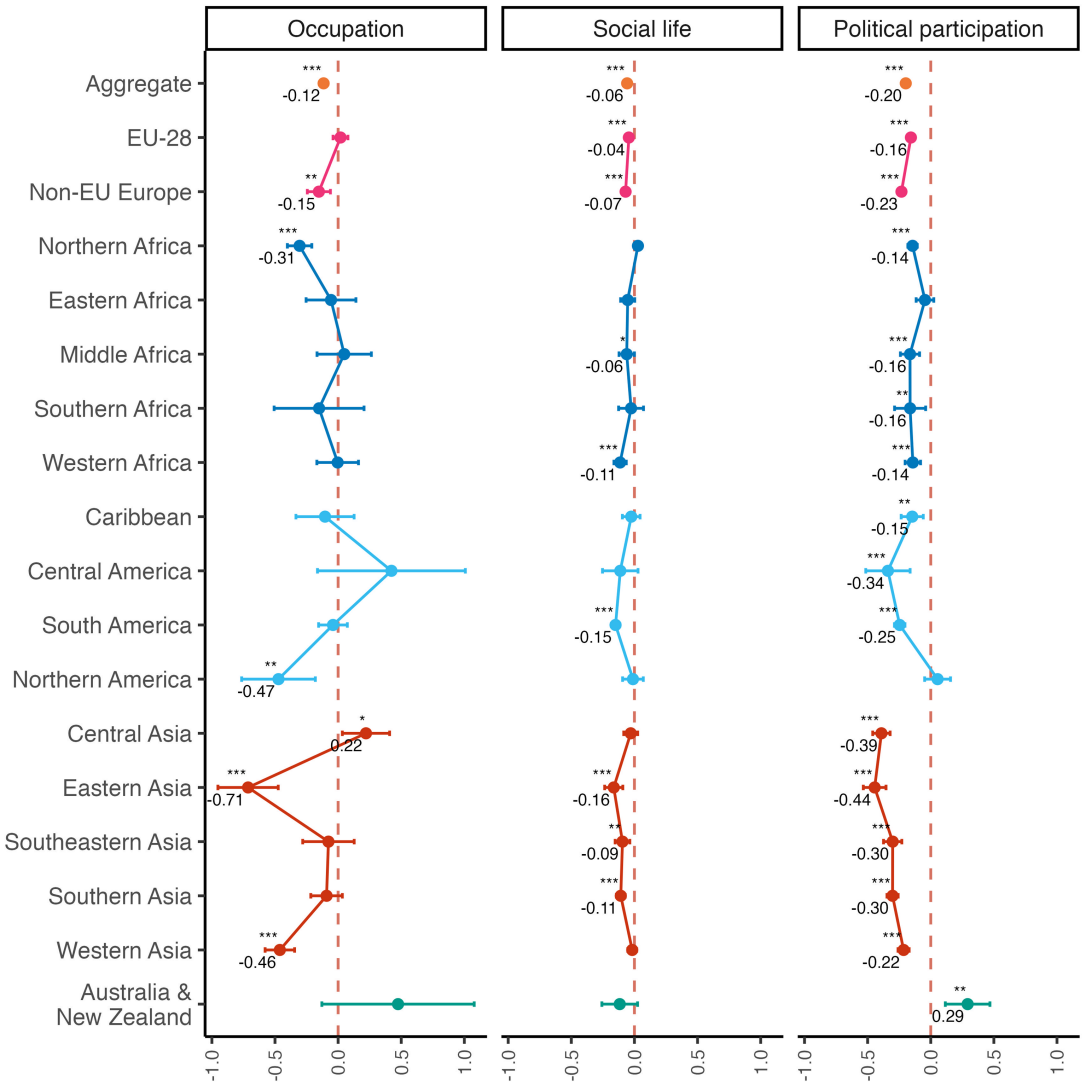
Effect of being foreign-born on integration indicators grouped by region of birth. Natives are the reference group. Printed values represent independent variable coefficients. Non-printed values indicate non-significant results (*p* > 0.05). ***p < 0.001; **p < 0.01; *p < 0.05.

We first examined whether immigrants and natives differed in the integration outcomes we obtained, and reproduced previous findings on migration (see Table 3). In our sample, immigrants were less likely to be employed [OR = 0.90, 95% CI (0.87, 0.93), *p* = 0.001; see Ballarino and Panichella (2015) and Fleischmann and Dronkers (2010)], had a less active social life [*β* = −0.06, 95% CI (−0.07, −0.05), *p* < 0.001; see Koelet and de Valk (2016) and ten Kate et al. (2020)], and were less politically engaged [*β* = −0.17, 95% CI (−0.19, −0.16), *p* < 0.001; see Giugni and Morales (2011)] than natives. Figure 2 displays these aggregate results, as well as the corresponding effects broken down by immigrants’ regions of origin, in order to represent how these integration differences vary across these regions—note that, while occupational status is reported as odds ratio in Table 3, in Figure 2 it is reported as estimates for a better visualization. Overall, foreign-born individuals are consistently worse off than natives for all indicators in most regions, with only two reversals (i.e., Australians and New Zealanders in the political participation dimension and Central Asians in occupational status, both being slightly better off than natives). Notably, the effect on political participation exhibits the greatest robustness to regions of origin—with a significant negative effect emerging in 14 out of the 17 regions of origin.

**Table 3:** Logistic and linear mixed-effects models of integration outcomes, predicted by foreign-born background.

	Occupational status		Social life		Political participation	
	Odds ratio	*p*	*β*	*p*	*β*	*p*
Foreign-born	0.90 [0.87, 0.93]	< 0.001	−0.06 [−0.07, −0.05]	< 0.001	−0.17 [−0.19, −0.16]	< 0.001
Age	1.06 [1.05, 1.07]	< 0.001	−0.05 [−0.06, −0.05]	< 0.001	0.04 [0.03, 0.04]	< 0.001
Gender: female	0.55 [0.54, 0.56]	< 0.001	−0.04 [−0.04, −0.03]	< 0.001	−0.02 [−0.03, −0.01]	< 0.001
Education	1.71 [1.69, 1.73]	< 0.001	0.05 [0.04, 0.05]	< 0.001	0.24 [0.24, 0.25]	< 0.001
Intercept	2.60 [2.35, 2.88]	< 0.001	0.04 [−0.01, 0.08]	0.12	−0.06 [−0.17, 0.05]	0.26
AIC	224,463		586,946		728,918	
Marginal Pseudo-*R*^2^/*R*^2^	0.09		0.01		0.06	
Conditional Pseudo-*R*^2^/*R*^2^	0.11		0.04		0.14	

**Figure 3:**
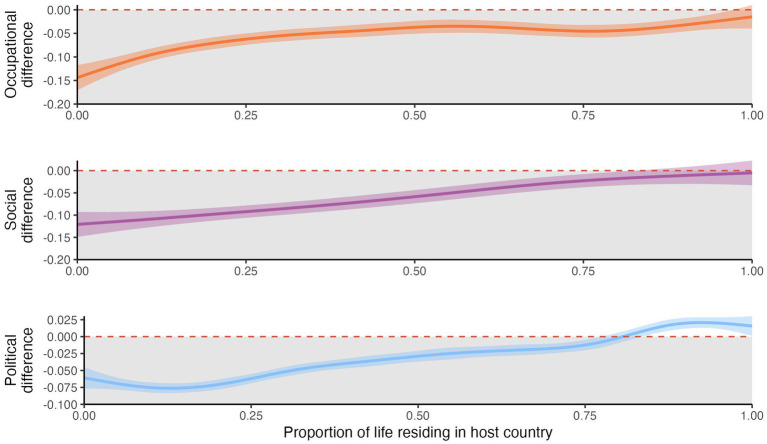
Reduction of the integration gap between natives and foreign-borns as they spend a larger proportion of their life in the host country.

Next, we asked whether the magnitude of the integration gaps depended on the proportion of life that immigrants had lived in the host country, thus providing three independent tests of convergence with natives—i.e., whether immigrants who spend a larger fraction of their lives in the host country approximate natives’ levels of integration across domains. Figure 3 plots the difference between immigrants and their reference group (composed of natives of their same gender, country of residence, and five-year age bracket) on each measure of integration (on the y-axis), against the proportion of their lives residing in the host country—ranging from 0 if the person is a newly-arrived to 1 if they have spent their whole life in the host country. Proportion of life in the host country has a positive effect for occupational integration [*β* = 0.069, 95% CI (0.053, 0.084), *t* = 8.68], social integration [*β* = 0.048, 95% CI (0.032, 0.063), *t* = 5.99], and political participation [*β* = 0.144, 95% CI (0.129, 0.159), *t* = 18.83], all *p*s < 0.001. These results provide evidence that the integration gap is largest for newly-arrived immigrants, and that immigrants tend to converge with natives for the three indicators here studied as they spend a larger fraction of their lives in the host country.

### Distribution of values

**Figure 4:**
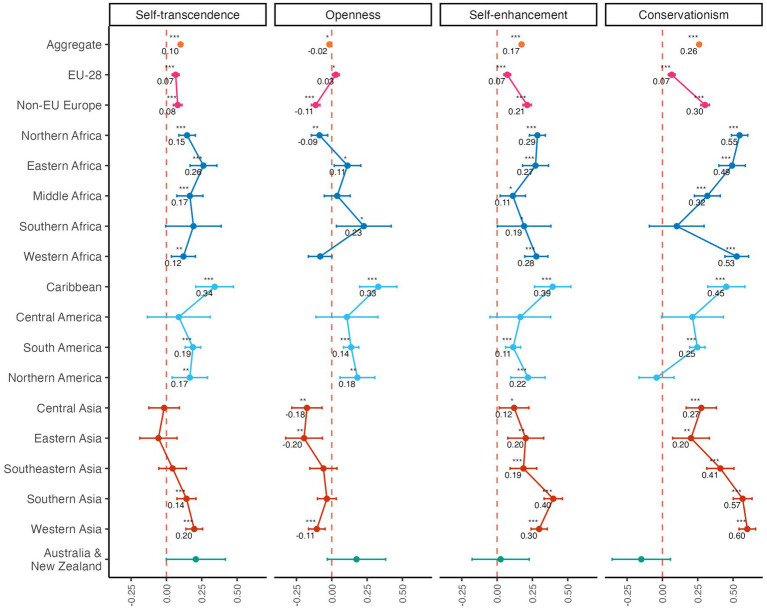
Effect of being foreign-born on Schwartz (1992) values grouped by region of birth. Natives are the reference group. Printed values represent independent variable coefficients. Non-printed values indicate non-significant results (*p* > 0.05). ***p < 0.001; **p < 0.01; *p < 0.05.

Having replicated previous evidence of integration gaps, and shown that these gaps diminish as immigrants spend a larger portion of their lives in the host country, we then tested whether immigrants and natives differ in their endorsement of human values while controlling for gender, age, and educational attainment. As shown in Table 4, the effect of being foreign-born was significant in all four models: Relative to natives, immigrants reported greater self-transcendence [*β* = 0.05, 95% CI (0.03, 0.06)], and especially self-enhancement [*β* = 0.24, 95% CI (0.22, 0.25)] and conservation [*β* = 0.33, 95% CI (0.31, 0.34)], all *p*s < 0.001, while reporting slightly lower levels of openness [*β* = −0.02, 95% CI (−0.03, −0.00), *p* = 0.026]; thus reproducing previous results (Czymara and Eisentraut, 2020). Figure 4 displays subgroup analyses by region of birth: the effects of being foreign-born on self-transcendence, self-enhancement and conservation values are present across most regions of origin. By contrast, the corresponding effect on openness values was heterogeneous across regions of origin–going from negative for immigrants of Asian origin to positive for those of American origin. Supplementary Analysis 5 reports the pattern of variation in values across countries of residence.

**Table 4:** Linear mixed-effects models of Schwartz’s human values.

	Self-transcendence		Openness		Self-enhancement		Conservation	
	*β*	*p*	*β*	*p*	*β*	*p*	*β*	*p*
Foreign-born	0.09 [0.08, 0.10]	< 0.001	−0.02 [−0.03, −0.00]	0.026	0.24 [0.22, 0.25]	< 0.001	0.33 [0.31, 0.34]	< 0.001
Age	0.04 [0.04, 0.05]	< 0.001	−0.25 [−0.26, −0.25]	< 0.001	−0.18 [−0.19, −0.18]	< 0.001	0.14 [0.13, 0.14]	< 0.001
Gender: Female	0.22 [0.21, 0.23]	< 0.001	−0.15 [−0.15, −0.14]	< 0.001	−0.19 [−0.20, −0.19]	< 0.001	0.12 [0.11, 0.13]	< 0.001
Education	0.09 [0.08, 0.09]	< 0.001	0.07 [0.07, 0.08]	< 0.001	0.04 [0.03, 0.04]	< 0.001	−0.10 [−0.11, −0.10]	< 0.001
Intercept	−0.23 [−0.35, −0.12]	0.001	0.09 [0.01, 0.17]	0.028	0.23 [0.10, 0.36]	0.002	−0.03 [−0.14, 0.08]	0.63
AIC	741,977		734,591		720,170		726,853	
Marginal Pseudo-*R*^2^/*R*^2^	0.02		0.08		0.05		0.04	
Conditional Pseudo-R^2^/R^2^	0.12		0.12		0.17		0.13	

**Figure 5:**
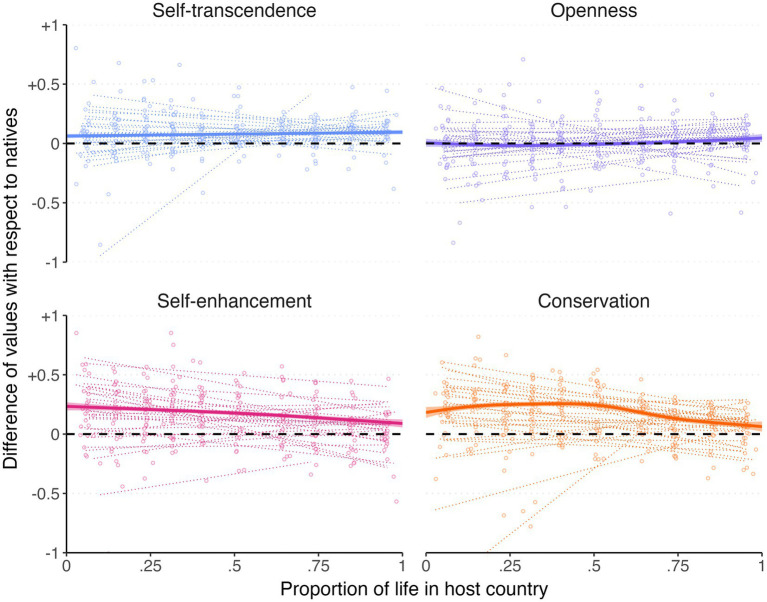
Standardized mean difference between foreign-born participants’ values by proportion of life in the host country. Jittered dots represent the mean values in each decile and country, and dotted trend lines display the linear trend for individual countries.

We also calculated the difference in moral values between each immigrant and the mean value in their native reference group, which we defined by grouping natives by 5-year age brackets, gender, and host country. Figure 5 displays the trajectory of foreign-born individuals’ values relative to the reference group, revealing a negative effect of proportion of life in the host country (i.e., toward assimilation) for self-enhancement [*β* = −0.082, 95% CI (−0.098, −0.067), *t* = −10.37] and conservation values [*β* = −0.105, 95% CI (−0.121, −0.090), *t* = −13.43], both *p*s < 0.001, and an opposite but considerably smaller effect on openness values [*β* = 0.027, 95% CI (0.011, 0.042), *t* = 3.40, *p* = 0.001] and self-transcendence values [*β* = 0.020, 95% CI (0.004, 0.036), *t* = 2.51, *p* = 0.012].

### Associations between values and integration indicators

Taking into consideration the mean differences in human values between natives and immigrants, and the evolution of these differences among immigrants as a function of the proportion of their lives spent in the host country (suggesting partial assimilation), our next analysis focuses on the relationship between human values and indicators of integration. The analysis reported in this section draws on the entire sample (i.e., both natives and immigrants), and therefore ought to be interpreted as describing the relationship between citizens’ values and life outcomes in the occupational, social, and political domains. We ran a logistic regression for the dichotomous occupational status measure, and linear regressions for the measures of social and political integration (see Table 5).

**Table 5:** Logistic and linear mixed-effects models of integration outcomes, predicted by groups of values.

	Occupational status		Social life		Political participation	
	Odds Ratio	*p*	*β*	*p*	*β*	*p*
Self-transcendence	0.96 [0.95, 0.98]	< 0.001	0.008 [0.003, 0.014]	0.002	0.160 [0.155, 0.165]	< 0.001
Openness	1.01 [0.99, 1.02]	0.38	0.146 [0.141, 0.152]	< 0.001	0.078 [0.073, 0.083]	< 0.001
Self-enhancement	1.10 [1.09, 1.12]	< 0.001	−0.001 [−0.007, 0.004]	0.58	−0.050 [−0.055, −0.045]	< 0.001
Conservationism	0.98 [0.96, 0.99]	< 0.001	−0.053 [−0.058, −0.048]	< 0.001	−0.168 [−0.173, −0.163]	< 0.001
Gender: Female	0.56 [0.55, 0.57]	< 0.001	−0.031 [−0.039, −0.022]	< 0.001	−0.035 [−0.043, −0.028]	< 0.001
Age	1.13 [1.12, 1.15]	< 0.001	−0.038 [−0.042, −0.034]	< 0.001	0.055 [0.051, 0.059]	< 0.001
Education	1.83 [1.81, 1.85]	< 0.001	0.045 [0.041, 0.049]	< 0.001	0.189 [0.184, 0.194]	< 0.001
Intercept	2.70 [2.42, 3.00]	< 0.001	0.049 [−0.013, 0.111]	0.13	0.025 [−0.063, 0.112]	0.59
AIC	258,980		509,165		−30,911	
Marginal Pseudo-*R*^2^/*R*^2^	0.12		0.03		0.09	
Conditional Pseudo-*R*^2^/*R*^2^	0.14		0.06		0.14	

Occupational status was unrelated to openness values (*p* = 0.38), positively associated with self-enhancement [*OR* = 1.10, 95% CI (1.09, 1.12)], and negatively related to self-transcendence [*OR* = 0.96, 95% CI (0.95, 0.98)] and conservationism values [*OR* = 0.98, 95% CI (0.96, 0.99); all *p*s < 0.001]. Meanwhile, social integration was positively related to self-transcendence [*β* = 0.008, 95% CI (0.003, 0.014), *p* = 0.002] and openness [*β* = 0.146, 95% CI (0.141, 0.152), *p* < 0.001], unrelated to self-enhancement [*β* = −0.001, 95% CI (−0.007, 0.004), *p* = 0.58] and negatively related to conservationism [*β* = −0.053, 95% CI (−0.058, −0.048), *p* < 0.001]. Lastly, political participation was associated positively with self-transcendence [*β* = 0.160, 95% CI (0.155, 0.165)] and openness [*β* = 0.078, 95% CI (0.073, 0.083)], and negatively with self-enhancement [*β* = −0.050, 95% CI (−0.055, −0.045)] and conservationism [*β* = −0.168, 95% CI (−0.173, −0.163)]; all *p*s < 0.001. These results support the idea that an individual’s values are associated with various life outcomes, such as the likelihood of employment, and their degree of social and political engagement.

### Deconfounding the effects on integration

**Figure 6:**
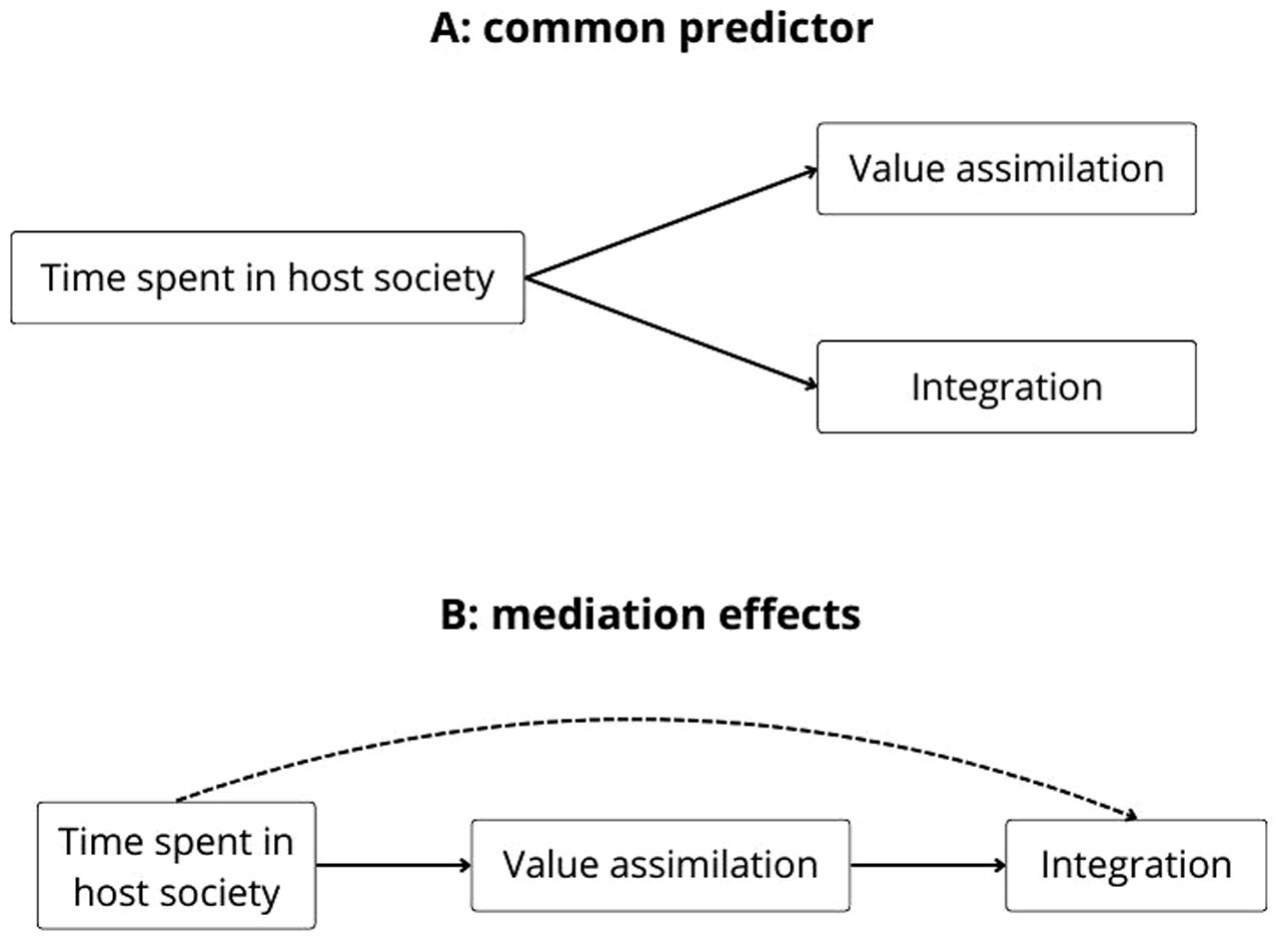
Plausible Models of Integration and Value Assimilation: **(A)** Common Predictor Model and **(B)** Model with Mediation Effects.

The previous analyses showed that immigrants and natives differ in their values and that values are related to positive life outcomes among both natives and immigrants. We have also shown that immigrants tend to assimilate as they spend a larger fraction of their lives in the host society. This raises the possibility that the relationship between value assimilation and integration is confounded by the proportion of immigrants’ lives spent in the host society. Specifically, one model would predict that as immigrants acquire a host society’s values over time, doing so in turn positively impacts integration—as predicted by assimilationist perspectives (see Figure 6B). Alternatively, integration and assimilation may be associated via their common cause, i.e., time spent in the host society (see Figure 6A). A third alternative, i.e., that integration mediates the effects of a foreign-born background on value assimilation, is examined in Supplementary Analysis 3.

To arbitrate between these competing models of integration and value assimilation, we stratified by the potential confound (i.e., time spent in the host society) in the subset of foreign-born participants (Tripepi et al., 2010). We created deciles of *proportion of life in the host society* and re-ran the above analyses (regressing integration outcomes on human values) separately in each stratum. The reasoning behind this deconfounding approach is the following: By drastically reducing the variance in the potential confounder (proportion of life in host society) *within* deciles, the relation between assimilation and integration should be substantially attenuated or even absent according to model A (in which the third variable is a common predictor). Yet according to model B (in which value assimilation mediates the relationship between time spent in the host society and integration), the independent relation between value assimilation and integration ought to persist—when holding constant time spent in the host society.

**Figure 7:**
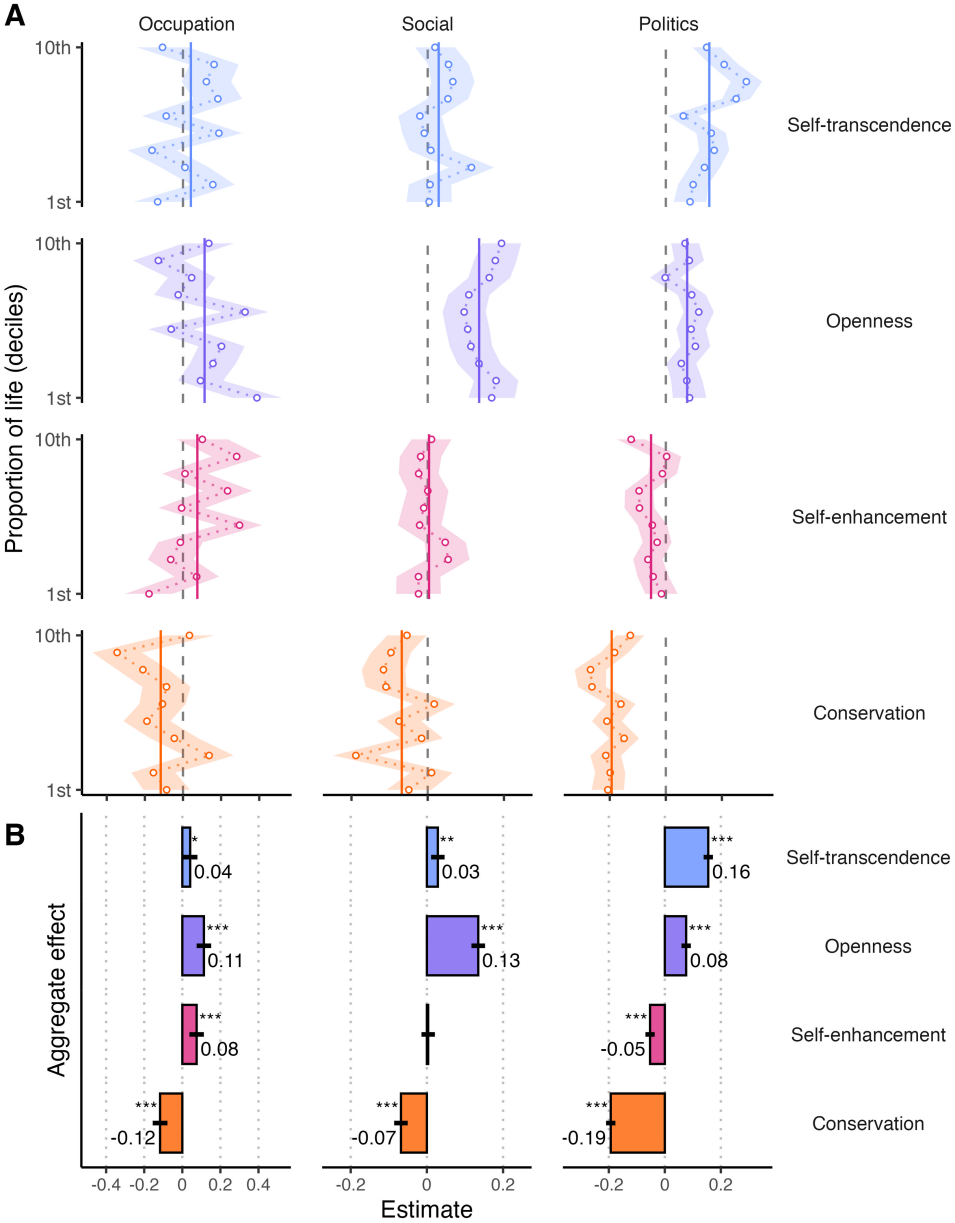
Effect of Schwartz’s human values on integration outcomes **(A)** for each decile of proportion of life in the host country and **(B)** in the aggregate. Printed values represent independent variable coefficients. Non-printed values indicate non-significant results (*p* > 0.05). ***p < 0.001; **p < 0.01, *p < 0.05.

Results of the stratified analyses are displayed in Figure 7A, and overall estimates of the deconfounded effects of human values are shown in Figure 7B. Self-transcendence positively relates to political [*β* = 0.16, 95% CI (0.15, 0.18), *p* < 0.001], social [*β* = 0.03, 95% CI (0.01, 0.05), *p* = 0.001], and also occupational integration to a lesser extent [*OR* = 1.04, 95% CI (1.00, 1.08), *p* = 0.031]. Openness values had positive effects on occupational [*OR* = 1.12, 95% CI (1.07, 1.16)], social [*β* = 0.14, 95% CI (0.12, 0.16)] and political integration [*β* = 0.08, 95% CI (0.07, 0.10)], all *p*s < 0.001. Self-enhancement values had no effect on social integration (*p* = 0.96), a small positive effect on occupation [*OR* = 1.07, 95% CI (1.03, 1.11), *p* = 0.001], and a small negative effect on political participation [*β* = −0.06, 95% CI (−0.08, −0.05), *p* < 0.001]. Meanwhile, conservation values had consistently negative effects on integration [occupation: *OR* = 0.90, 95% CI (0.87, 0.94); social: *β* = −0.07, 95% CI (−0.09, −0.06)], which were strongest for political integration [*β* = −0.20, 95% CI (−0.22, −0.19)], all *p*s < 0.001.

These results provide evidence of multiple associations between value assimilation and integration in other domains. Specifically, self-transcendence and openness values predict improved integration to some degree, self-enhancement values reveal mixed effects across domains of integration, and conservation values predict a worse integration, especially in the political plain. Although these results provide support for model B in Figure 6, and the stratification method ruled out the confounding effects of a common predictor—i.e., time spent in host society—, they should not be interpreted as providing causal evidence of an influence of value assimilation on integration. Strictly speaking, they provide evidence of significant associations between value assimilation and broader integration that are independent from the effects of time residing in the host country.

### Do differences in human values explain the integration gap?

The previous sections established that (i) natives and immigrants differ in their moral values, and that (ii) moral values appear to predict integration success in at least some aspects. In this section, we will ask whether and how the observed differences between natives’ and immigrants’ moral values explain the integration gap. To this end, we conducted a parallel mediation analysis in the *lavaan* R package (Rosseel, 2012), with respondents nested within host countries in a hierarchical model, allowing intercept variance across countries. As shown in Table 6, these analyses revealed that the negative effect of being foreign-born on integration outcomes was primarily direct (with direct/total effect proportions in the range between 58 and 105%), although several indirect paths were statistically significant as well. Most importantly, the indirect effects via conservation values appeared to account for 15.5% (for social integration) and 53.8% (for political participation) of the integration gap between immigrants and natives, though only 4% of the integration gap in occupational status. Subgroup analyses for each region of origin can be found in Supplementary Analysis 2.

**Table 6:** Multiple mediation analyses: direct and indirect effects of immigrant (versus native) origin via Schwartz’s human values.

	Occupational status			Social integration			Political participation		
	*B*	*z*	*p*	*B*	*z*	*p*	*B*	*z*	*p*
Total effect of foreign-born	−0.033	−9.35	< 0.001	−0.067	−11.42	< 0.001	−0.020	−11.18	< 0.001

	**Prop.**	***z***	***p***	**Prop.**	***z***	***p***	**Prop.**	***z***	***p***
Direct effect of Foreign-born	1.049	86.36	< 0.001	0.822	40.37	< 0.001	0.579	14.60	< 0.001
Indirect effect: self-transcendence	0.024	4.92	< 0.001	−0.012	−3.63	< 0.001	−0.229	−8.19	< 0.001
Indirect effect: openness	0.002	1.76	0.079	0.027	2.20	0.028	0.011	1.55	0.12
Indirect effect: self-enhancement	−0.114	−7.69	< 0.001	0.008	1.44	0.15	0.101	9.03	< 0.001
Indirect effect: conservation	0.038	2.36	< 0.001	0.155	9.59	< 0.001	0.538	11.07	< 0.001
AIC	2,321,289			1,423,450			1,175,404		
*R* ^2^	0.08			0.03			0.11		

## Discussion

The pursuit of integration involves ensuring that immigrants thrive in the host society across numerous facets of life. Unfortunately, immigrants frequently encounter greater difficulties than native citizens in their pursuit of employment, social capital, and political participation (Koelet and de Valk, 2016; Ballarino and Panichella, 2015; Giugni and Morales, 2011), among other aspects. In this paper, we show how newly arrived immigrants significantly differ from natives in their self-reported values, and how this gap gradually diminishes as immigrants spend a greater share of their lives in the host society. This assimilation of values accompanies a reduction in the integration gap between immigrants and natives for all indicators here studied. The parallel trajectories of immigrants’ moral values and their integration is part of the rationale that many European states offer when demanding civic integration among their immigrant populations. Nevertheless, this claim rests on the assumption that assimilation of native moral values actually facilitates immigrants’ integration in the other domains—such as work, social life and politics, and raises an empirical question: Does immigrants’ moral ‘maladaptation’ to the host society hinder their overall integration, as implied by the assimilationist model?

By deconfounding the relationship between moral values and integration outcomes from their potential common predictor (i.e., the proportion of an immigrant’s life spent in the host country), we obtained evidence that moral values predict common integration indicators including employment status, being more socially active, and political engagement. Given that immigrants and natives tend to differ in moral values on average, and moral values predict integration outcomes, our final analyses asked whether the observed differences in moral values might explain the integration gap between immigrants and natives.

A mediation analysis suggested that the indirect effects on integration via moral values accounted for a negligible fraction of the total effect of non-native origin, with one notable exception: Namely, the tendency for immigrants to more strongly endorse conservation values (i.e., an orientation toward tradition, conformity, and security) appeared to constitute a barrier to integration in the political domain. This result coheres with evidence that political integration is the most demanding aspect of immigrants’ integration into a host country (Ramakrishnan, 2006) and is most strongly linked to values (Abdallah, 2023), potentially due to a combination of legal obstacles (Cinalli and Giugni, 2011), conflicts with individuals’ loyalty to their country of origin (Chaudhary, 2018; Morales and Morariu, 2011), and the sense that political participation is an optional step in the adaptation to a host society, by comparison to employment or social connectedness (Giugni and Morales, 2011).

Overall, our results suggest that values only weakly associate with certain aspects of the integration gap between natives and immigrants—a finding that calls into question whether the proliferation of civic integration policies in Europe is justified on the grounds of its efficiency in reducing the immigrant integration gap.

### Limitations and future lines of research

First, although occupational status, social activity, and political participation are central indicators of successful integration for immigrants, further dimensions, such as linguistic, psychological, and navigational integration can also play an important role (Harder et al., 2018). Our data source, however, did not allow us to establish how immigrants’ fare on these additional dimensions—leaving open whether future research will extend our present findings beyond the dimensions we examined here. Additionally, the decision to code immigrants as foreign-born disregards subtleties within migrant communities—including second generation immigrants, who qualify as natives in our coding system—(see Portes et al. (2005) and Portes and Zhou (1993) for differences between natives and second generation immigrants)—and the so-called ‘decimal generations’ (Oropesa and Landale, 1997), who are coded as immigrants even though they have socialized in the host country during most of their childhood. Furthermore, although the construction of our variables was validated by previous research, we acknowledge that they lack the nuance to capture the complexity of immigrants’ integration processes. For instance, occupation does not capture whether respondents’ primary occupation accords with their expertise and training, or even if they have freely decided not to work for personal reasons; social integration indicators only capture whether immigrants have a social network in their host country, but does not distinguish social networks composed of other immigrants (implying segregation) vs. natives (implying integration); and political participation indicators only refer to behavioral manifestations of it, while neglecting other relevant aspects of political integration such as attitudes toward national politics, electoral participation (when possible), or the importance given to citizenship acquisition.

Second, and in line with the previously expressed limitation, although all models statistically controlled for the country of *residence* and some also consider regions of *origin* (including further analyses in Supplementary Analyses 2, 5), there are other relevant aspects of immigration, such as legal status or reasons to migrate, that are beyond the scope of the present work, yet may crucially influence integration.

Third, analyses of cross-sectional data are limited in their ability to draw causal conclusions, even with recent developments in causal inference methods (Pearl, 2010). In light of the possibility of unobserved confounds, our analyses to deconfound the relationship between the adoption of moral values and successful integration should be interpreted with caution. Confirmation of a causal relationship between value assimilation and integration a waits convergent evidence from longitudinal and/or quasi-experimental studies that may provide more decisive evidence of causation.

## Conclusion

This paper explores how value assimilation is associated with immigrants’ broader integration along the dimensions of occupational status, social life, and political participation. By analysing the effects of different sets of values on integration indicators among the foreign-born population in the EU countries present in the European Social Survey, we have found that, although values are significantly related to the integration outcomes we examined, these effects only account for a negligible portion of the integration gap.

Many liberal democracies in Europe have been increasingly implementing formal requirements and integration policies that focus on the value dimension—the so-called civic integration—, on top of other more typical demands such as residence periods, socioeconomic integration, or language acquisition, among others (Perchinig, 2012). These civic integration measures are aimed at helping the immigrant population in integrating better in the host society and thus enabling them to become active citizens, although in most cases they are framed as civic integration courses, tests, or interviews. Our results contribute to the understanding of these requirements of citizenship by providing transnational evidence that civic integration initiatives may not provide efficient means to ensure immigrants’ overall integration, therefore potentially raising new questions about the justification and legitimacy of such policies.

This recent turn toward civic integrationist frameworks in Europe during the past decades stirs debate about a return to assimilationist views (Joppke, 2017; Goodman, 2014). Some authors have pointed out that cultural assimilation is often the first step in integration processes (Gordon, 1964). Others have argued that it enables social cohesion at a macro level, and also individually benefits immigrants who would otherwise remain isolated within the host society [(Miller, 2009), but see Larin (2020)]. Empirical research on value assimilation has focused inordinately on the first question (whether value congruence confers certain macro-level benefits, e.g., protecting societal stability as a whole) while comparatively neglecting the second [for recent exceptions, see Pronin et al. (2004) and Fuchs and von Scheve (2023)]. In our present study we turned our attention to the question of whether assimilation benefits immigrants’ integration processes. Gathering evidence from ten waves of the European Social Survey, we found that the relationship between values and integration was modest overall, accounting for a negligible fraction of the integration gap—with the exception of conservation values, which appeared to hinder immigrants’ political involvement. In sum, setting aside the delicate question of whether demanding conformity to host societal values is politically desirable or even normatively defensible, our present research casts doubt on the empirical presupposition that value assimilation helps to narrow the integration gap between immigrants and natives throughout Europe.

## Data Availability

The original contributions presented in the study are included in the article/Supplementary material, further inquiries can be directed to the corresponding author/s.
