# Supplementary material for: Integration gaps persist despite immigrants’ value assimilation: evidence from the European Social Survey
Source: Front Sociol. 2025 Apr 1;10:1504127. doi: 10.3389/fsoc.2025.1504127 (PMC11997842; doi:10.3389/fsoc.2025.1504127)
Supplement: Supplementary file 1 [file Data_Sheet_1.docx]

Supplementary Material

# Supplementary analysis 1: Models by ESS rounds

## SA Tables and figures


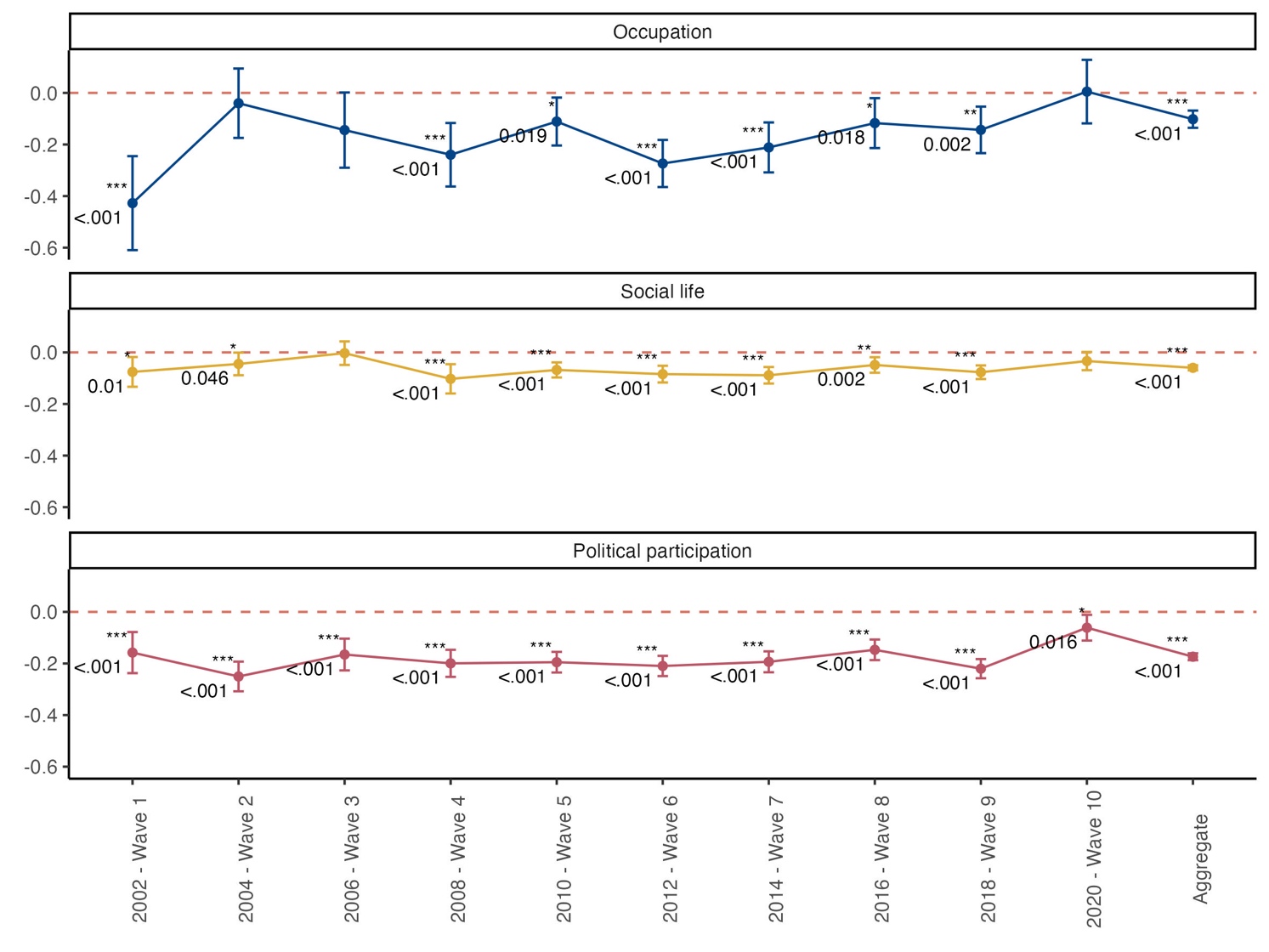


**Supplementary Figure 1.1.** Effect of being foreign-born on integration indicators for each separate ESS round as well as the aggregate of all 10 rounds. Printed values represent p-values for each individual model. Non-printed values indicate non-significant results (p > .05).


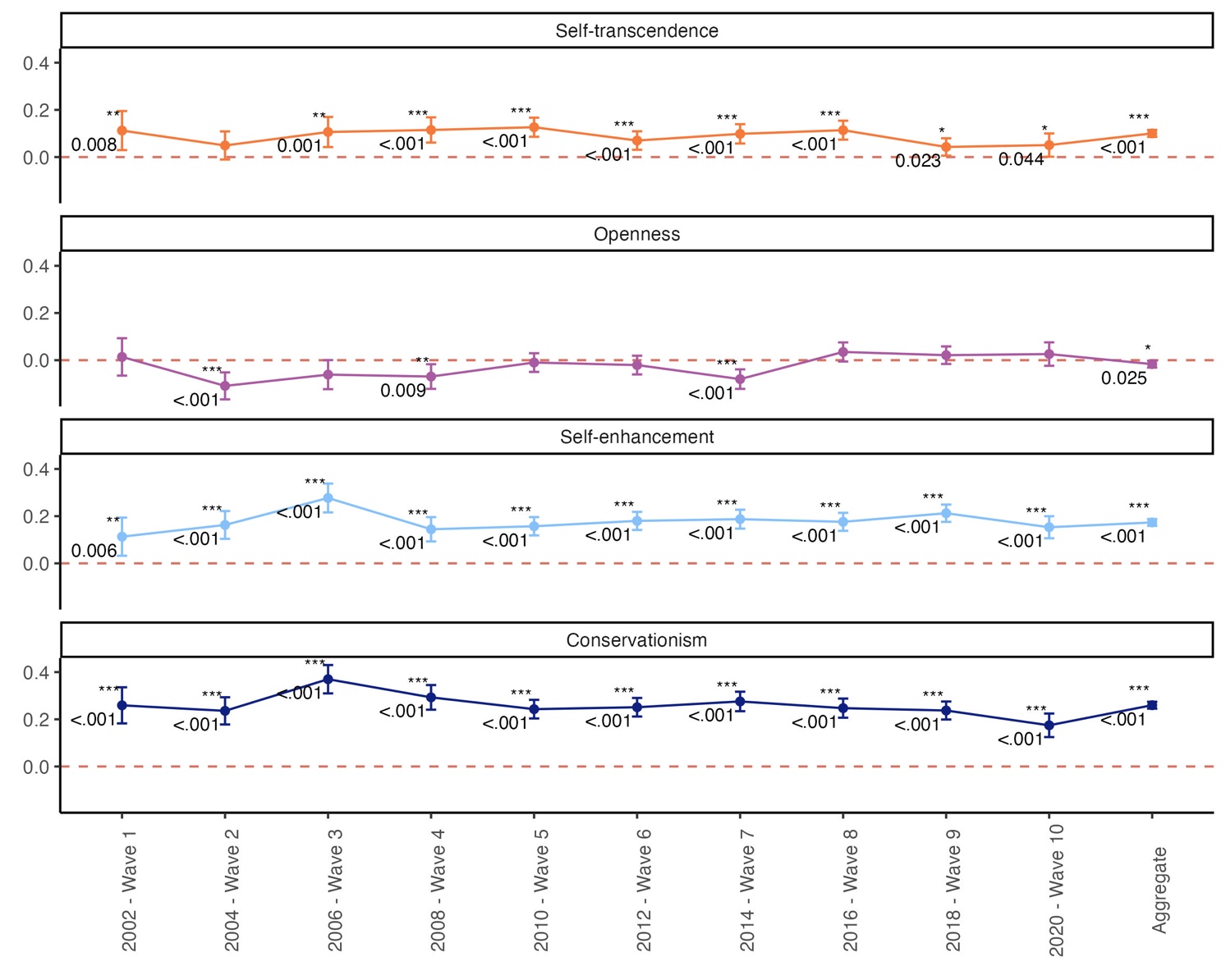


**Supplementary Figure 1.2.** Effect of being foreign-born on groups of values according to Schwartz (1992) for each separate ESS round as well as the aggregate of all 10 rounds. Printed values represent p-values for each individual model. Non-printed values indicate non-significant results (p>.05).


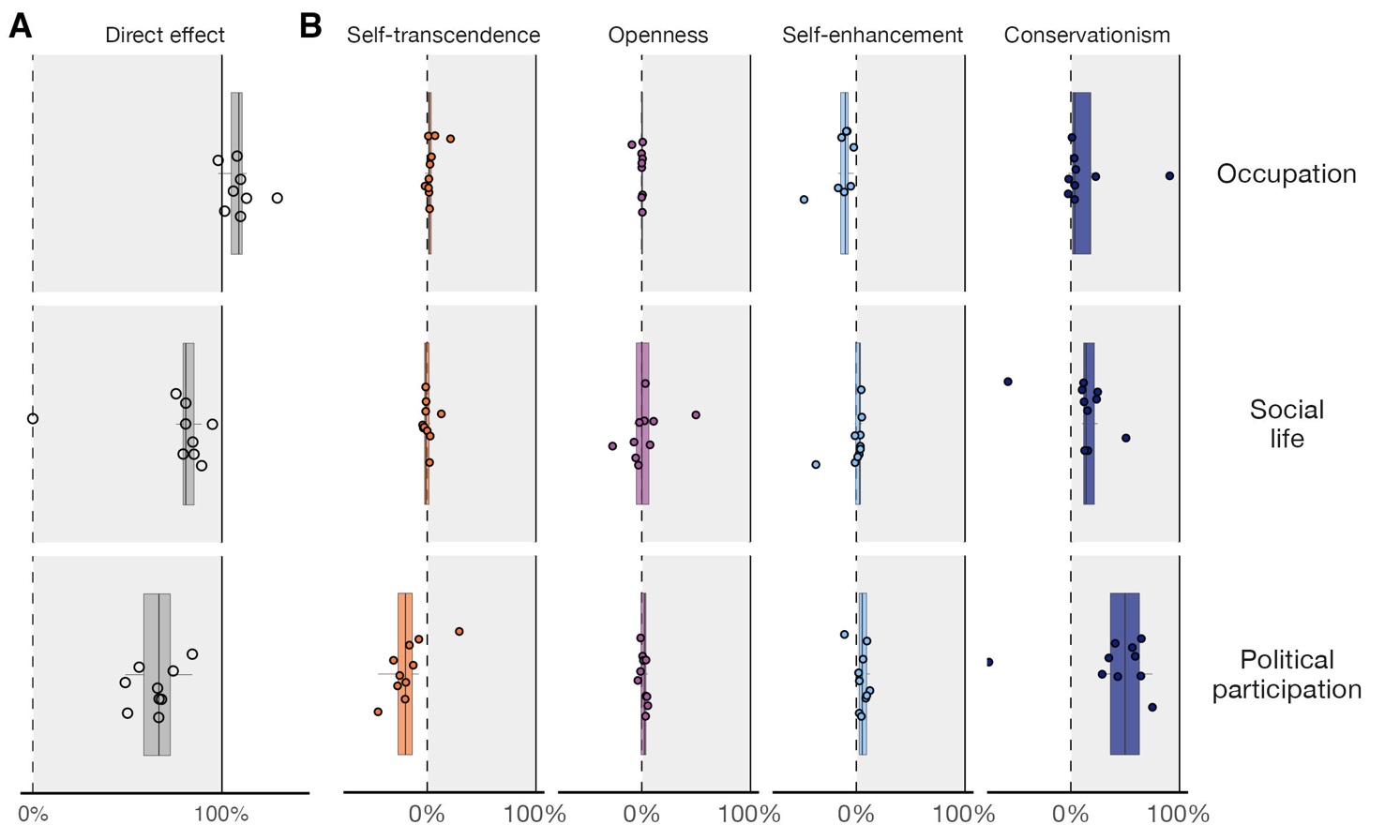


**Supplementary Figure 1.3.** A) Direct effect of being foreign-born on integration indicators as a proportion of the total effect. B) Indirect effect of each group of values on integration indicators as a proportion of the total effect. Each point represents a different ESS round and boxplots represent the aggregate.

## Discussion

Here we reproduce the main analyses shown in the main manuscript comparing all ten available ESS rounds. To justify aggregating rounds from different years, with these analyses we aim to detect whether the effects we look for are stable across each wave. Thus, we aim to solve potential confounds of the study year that might occur due to an evolution of our variables of interest throughout the almost two decades of ESS data.

Supplementary Figures 1.1 and 1.2 show that the effects of being foreign-born on our dependent (integration indicators) and independent (values) variables are stable across rounds, with no inversion of the direction of effects for any of the rounds. However, although all aggregate models offer significant results under the p<.05 threshold, some individual rounds are not significant. This can be due to a lack of statistical power for the independent samples, especially considering that the migrant population represents less than 10 per cent in all samples.

Supplementary Figure 1.3 additionally reproduces the effects of all mediation models for each separate round (represented by the dots) and the aggregate (boxplot). As observed, most rounds fall near the aggregate, with some outliers indicating unclear and non-significant results for a few individual rounds.

Altogether, we believe that these results can support the use of the aggregate of all ESS rounds, mainly for two reasons: first, because results are stable across all rounds and there is no evidence that the effect could be the inverse for any of the models; second, because a few individual rounds do not offer significant results, potentially and most likely due to a lack of statistical power that is solved after aggregating all rounds.

# Supplementary analysis 2: Differentiating immigrants’ regions of origin

## SA Tables and figures

| **Supplementary Table 2.1.** Number of respondents by birth region and ESS round. | | | | | | | | | | | |
| --- | --- | --- | --- | --- | --- | --- | --- | --- | --- | --- | --- |
|  | **ESS Round** | | | | | | | | | | **Aggregate (column %)** |
|  | **1**  **(2002)** | **2**  **(2004)** | **3**  **(2006)** | **4**  **(2008)** | **5**  **(2010)** | **6**  **(2012)** | **7**  **(2014)** | **8**  **(2016)** | **9**  **(2018)** | **10**  **(2020)** |  |
| **Native** | 21,727 | 24,404 | 22,345 | 29,252 | 27,354 | 26,919 | 21,859 | 22,573 | 25,293 | 19,308 | 241,034  (92.43) |
| **EU-28** | 564 | 841 | 557 | 722 | 821 | 918 | 786 | 825 | 914 | 476 | 7424  (2.85) |
| **Non-EU Europe** | 195 | 441 | 293 | 533 | 593 | 439 | 453 | 441 | 620 | 380 | 4388  (1.68) |
| **Northern Africa** | 68 | 63 | 109 | 101 | 107 | 147 | 122 | 134 | 155 | 114 | 1120  (0.43) |
| **Eastern Africa** | 46 | 27 | 37 | 40 | 64 | 66 | 60 | 44 | 74 | 33 | 491  (0.19) |
| **Middle Africa** | 21 | 44 | 47 | 44 | 47 | 59 | 50 | 44 | 54 | 63 | 473  (0.18) |
| **Southern Africa** | 8 | 8 | 15 | 16 | 7 | 19 | 17 | 6 | 21 | 12 | 129  (0.05) |
| **Western Africa** | 29 | 38 | 41 | 58 | 75 | 76 | 63 | 64 | 74 | 62 | 580  (0.22) |
| **Caribbean** | 7 | 15 | 21 | 26 | 24 | 24 | 30 | 30 | 31 | 21 | 229  (0.09) |
| **Central America** | 3 | 1 | 4 | 3 | 5 | 18 | 13 | 11 | 13 | 3 | 74  (0.03) |
| **South America** | 64 | 80 | 102 | 143 | 146 | 181 | 121 | 162 | 164 | 117 | 1280  (0.49) |
| **Northern America** | 16 | 32 | 41 | 31 | 38 | 39 | 27 | 31 | 37 | 18 | 310  (0.12) |
| **Central Asia** | 19 | 25 | 22 | 31 | 45 | 46 | 32 | 34 | 49 | 9 | 312  (0.12) |
| **Eastern Asia** | 19 | 14 | 12 | 21 | 30 | 46 | 36 | 10 | 38 | 14 | 240  (0.09) |
| **Southeastern Asia** | 29 | 43 | 38 | 41 | 52 | 50 | 47 | 52 | 51 | 26 | 429  (0.16) |
| **Southern Asia** | 54 | 63 | 69 | 61 | 111 | 128 | 143 | 107 | 152 | 65 | 953  (0.37) |
| **Western Asia** | 130 | 110 | 90 | 78 | 146 | 147 | 142 | 118 | 194 | 41 | 1196  (0.46) |
| **Australia & New Zealand** | 11 | 10 | 11 | 14 | 14 | 15 | 11 | 14 | 13 | 6 | 119  (0.05) |
| **Melanesia** | 1 | 1 | 0 | 0 | 0 | 1 | 0 | 1 | 0 | 0 | 4  (0.00) |
| **Micronesia** | 0 | 0 | 0 | 0 | 0 | 0 | 0 | 0 | 0 | 0 | 0  (0.00) |
| **Polynesia** | 0 | 0 | 0 | 0 | 0 | 0 | 1 | 1 | 0 | 0 | 2  (0.00) |
| **Total** | 23,011 | 26,260 | 23,854 | 31,215 | 29,679 | 29,338 | 24,013 | 24,702 | 27,947 | 20,768 | 260,787  (100.00) |

**
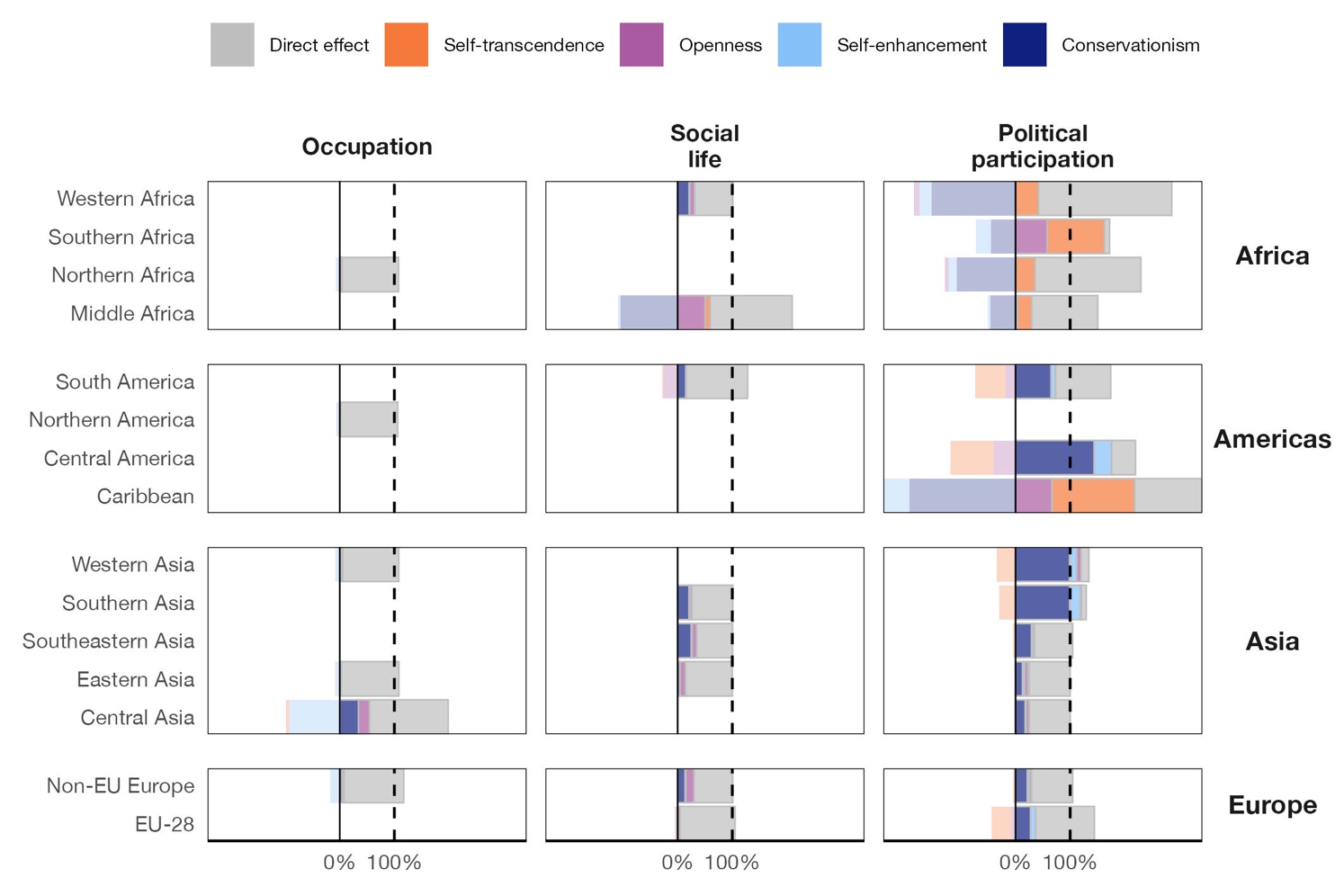
**

**Supplementary Figure 2.1.** Direct effect of being foreign-born and indirect effects of each group of values on integration indicators as percentages of the total effect. Non-significant total effects are not displayed.

## Discussion

These analyses aim to expand on whether effects of being foreign-born persist across different regions of origin. To do this, we have grouped immigrants’ declared country of birth according to the United Nations geoscheme, which divides all countries in 22 subregions. We have excluded participants whose country of birth was in the regions of Melanesia, Micronesia, or Polynesia, due to their negligible sample size which is not suitable for our models (see Supplementary Table 2.1). Additionally, European regions have being grouped in countries who belonged to the European Union as of 2020 (EU-28) and European countries who are not members of the EU. The rationale for this grouping is based on the fact that our sample only contains respondents who live in EU-28 countries, therefore being more affected by policies that apply differently to European and non-European citizens. In the end, our whole sample is assigned to one of 16 regions—note that the region of Australia and New Zealand is not represented due to its lack of significant total effects.

In Supplementary Figure 2.1, looking across regions, the negative effects of being foreign-born are primarily direct. The indirect effect via conservation on political participation appears to be strongest among immigrants of Western and Southern Asian, and Central and South American origins. Similarly, indirect effects via self-transcendence seem stronger for African and Caribbean origins. For occupational and social integration, these results reproduce the findings of the main manuscript: the model with indirect effects of values on these integration indicators does not offer a good fit and the largest effect is by far direct. On the other hand, political integration models in the aggregate had shown that conservation values did have considerable indirect effects. In the separate analyses, we can see that this holds true mainly for specific origins that are at the same time some of the largest groups in the overall sample of foreign-born respondents (see Supplementary Table 2.1), which can be the reason why this effect persists in the aggregate even though it being specific to only a few regions.

# Supplementary analysis 3: Opposite mediation models

## SA Tables and figures

| **Supplementary Table 3.1.** Multiple Mediation Analyses (opposite direction as in main manuscript): Direct and Indirect Effects of Immigrant (versus Native) background on Values via Integration Indicators | | | | | | | | | | | | |
| --- | --- | --- | --- | --- | --- | --- | --- | --- | --- | --- | --- | --- |
|  | **Self-transcendence**  **AIC = 1180734**  **R^2^ = .03** | | | **Openness**  **AIC = 1244360**  **R^2^ = .11** | | | **Self-enhancement**  **AIC = 1326520**  **R^2^ = .05** | | | **Conservationism**  **AIC = 1238586**  **R^2^ = .05** | | |
|  | ***B*** | ***z*** | ***p*** | ***B*** | ***z*** | ***p*** | ***B*** | ***z*** | ***p*** | ***B*** | ***z*** | ***p*** |
| *Total effect of Foreign-born* | 0.124 | 23.01 | <.001 | 0.009 | 1.39 | .17 | 0.069 | 9.21 | <.001 | 0.154 | 25.02 | <.001 |
|  | **prop.** | ***z*** | ***p*** | **prop.** | ***z*** | ***p*** | **prop.** | ***Z*** | ***p*** | **prop.** | ***z*** | ***p*** |
| *Direct effect of Foreign-born* | 1.098 | 156.75 | <.001 | 3.504 | 1.92 | .055 | 1.042 | 88.01 | <.001 | 0.913 | 168.55 | <.001 |
| *Indirect effect: Occupation* | 0.006 | 4.83 | <.001 | -0.130 | -1.36 | .18 | -0.050 | -6.32 | <.001 | -0.001 | -0.90 | .37 |
| *Indirect effect: Social life* | -0.016 | -8.40 | <.001 | -1.087 | -1.36 | .18 | -0.073 | -6.88 | <.001 | 0.010 | 7.00 | <.001 |
| *Indirect effect: Political participation* | -0.087 | -13.70 | <.001 | -1.286 | -1.38 | .17 | 0.081 | 7.54 | <.001 | 0.076 | 15.33 | <.001 |

## Discussion

These mediation analyses correspond to the model where the effect of being foreign-born on upholding certain values is causally mediated by the degree to which individuals have acquired integration in occupation, social life, and political participation—i.e., the opposite path tested in the main manuscript, where values mediated the effect on integration.

As seen in Supplementary Table 3.1, there are no significant effects for openness values—neither direct nor indirect—, while the rest of the models are mostly explained through direct effects (*p*s<.001). All integration indicators have significant indirect effects (*p*s<.001) on all values (except openness), apart from occupational status on conservationism values (*p*=.27). Nevertheless, indirect effects account for a negligible fraction of the total effect, with a maximum of 8.5% for political participation on self-transcendence values in negative terms.

Altogether, these results do not support the model where differences of values between natives and immigrants are mediated by the degree to which the latter are integrated, at least in terms of their occupational status, social life, and political participation. At best, the indirect effects of these indicators only account for a small fraction that is nonetheless significant under the *p* < .001 threshold.

# Supplementary analysis 4: Comparing measures of time spent in host country

## SA Tables and figures


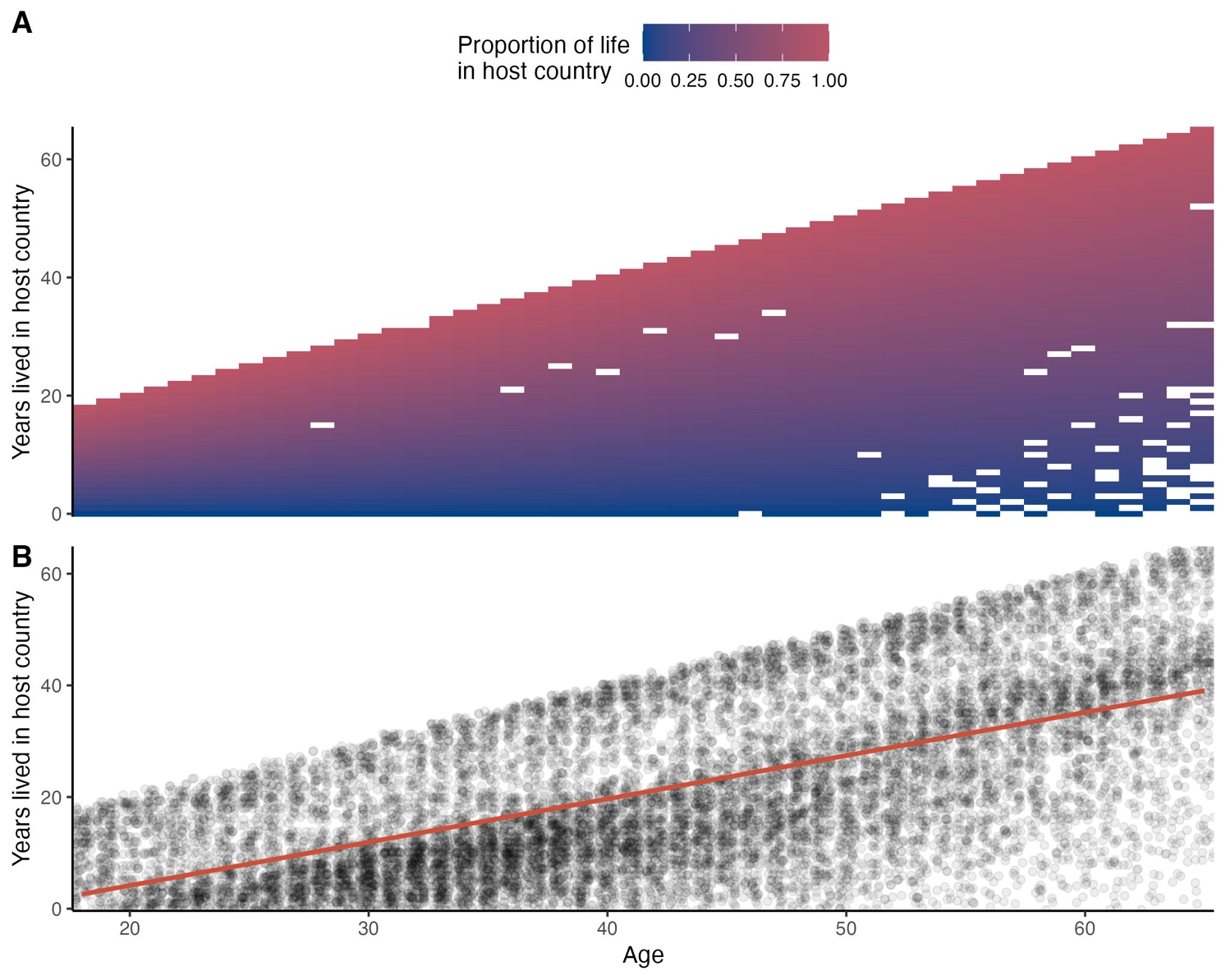


**Supplementary Figure 4.1.** Relation between immigrants’ age and the constructed variables of years and proportion of life lived in host country. A) Heat-map with colour gradient identifying individuals whose proportion of life in host country is equivalent; B) scatterplot with regression line representing the distribution of immigrant respondents.

## Discussion

The main manuscript makes use of the constructed variable of proportion of life as independent variable to explore whether value assimilation occurs, i.e., whether individuals who have lived a larger share of their lives are more similar to natives in their values. The decision of using this constructed variable is based on the variability of immigration backgrounds and socialisation dynamics. As can be seen in Supplementary Figure 4.1, there is a wide myriad of combinations that pose the challenge of comparing different individuals whose immigration process differs significantly.

Since in our main analyses we aim to measure whether processes of socialisation of immigrants in the host country predict their similarity in values to natives, this can be either represented by means of the amount of years or their share of life spent in it. However, the first option­­—i.e., using the amount of years—forces to compare individuals that, although having lived the same amount of years in the host country, have socialised in complete different ways in their countries of origin. For instance, a 20-year-old immigrant who arrived at 10 would be compared to a 60-year-old who arrived at 50. However, while the first person has been socialising in the host country for half of their life, the second has only thus lived for a sixth. As the figures show, using the proportion of life spent in the host country offers the advantage of comparing more similar socialisation backgrounds—e.g., a 20-year-old person who has spent the last 10 in the host country *versus* a 60-year-old who has spent the last 30 in the host country, therefore having both lived half of their lives in it. In other words, it is expectable that people who arrive to the host country at an older age will take more time to adapt and thus overcome the effects of a larger socialisation process that priorly happened in their country of origin.

# Supplementary Analysis 5: Value distribution by country

## SA Tables and figures

| **Supplementary Table 5.1.** Number of native- and foreign-born respondents by country | | | |
| --- | --- | --- | --- |
| **Country** | **Native (%)** | **Foreign-born (%)** | **Total** |
| **Austria - AT** | 8921 (90.8) | 901 (9.2) | 9822 (100.0) |
| **Belgium - BE** | 11405 (87.5) | 1635 (12.5) | 13040 (100.0) |
| **Bulgaria - BG** | 7947 (99.4) | 44 (0.6) | 7991 (100.0) |
| **Cyprus - CY** | 3405 (91.6) | 314 (8.4) | 3719 (100.0) |
| **Czechia - CZ** | 13384 (98.1) | 259 (1.9) | 13643 (100.0) |
| **Germany - DE** | 16773 (90.6) | 1749 (9.4) | 18522 (100.0) |
| **Denmark - DK** | 8385 (94.0) | 540 (6.0) | 8925 (100.0) |
| **Estonia - EE** | 10252 (87.5) | 1468 (12.5) | 11720 (100.0) |
| **Spain - ES** | 11144 (89.8) | 1270 (10.2) | 12414 (100.0) |
| **Finland - FI** | 12186 (96.4) | 459 (3.6) | 12645 (100.0) |
| **France - FR** | 12066 (90.4) | 1287 (9.6) | 13353 (100.0) |
| **United Kingdom - GB** | 12551 (88.0) | 1711 (12.0) | 14262 (100.0) |
| **Greece - GR** | 8348 (91.8) | 741 (8.2) | 9089 (100.0) |
| **Croatia - HR** | 4010 (91.8) | 360 (8.2) | 4370 (100.0) |
| **Hungary - HU** | 11362 (98.5) | 169 (1.5) | 11531 (100.0) |
| **Ireland - IE** | 12126 (83.7) | 2358 (16.3) | 14484 (100.0) |
| **Italy - IT** | 5331 (90.8) | 543 (9.2) | 5874 (100.0) |
| **Lithuania - LT** | 7321 (97.4) | 197 (2.6) | 7518 (100.0) |
| **Luxembourg - LU** | 829 (70.0) | 355 (30.0) | 1184 (100.0) |
| **Latvia - LV** | 1766 (90.7) | 181 (9.3) | 1947 (100.0) |
| **Netherlands - NL** | 12065 (91.3) | 1152 (8.7) | 13217 (100.0) |
| **Poland - PL** | 11275 (99.5) | 58 (0.5) | 11333 (100.0) |
| **Portugal - PT** | 10678 (92.0) | 935 (8.1) | 11613 (100.0) |
| **Romania - RO** | 1536 (99.6) | 6 (0.4) | 1542 (100.0) |
| **Sweden - SE** | 9253 (88.6) | 1187 (11.4) | 10440 (100.0) |
| **Slovenia - SI** | 8838 (91.9) | 778 (8.1) | 9616 (100.0) |
| **Slovakia - SK** | 7877 (98.3) | 139 (1.7) | 8016 (100.0) |


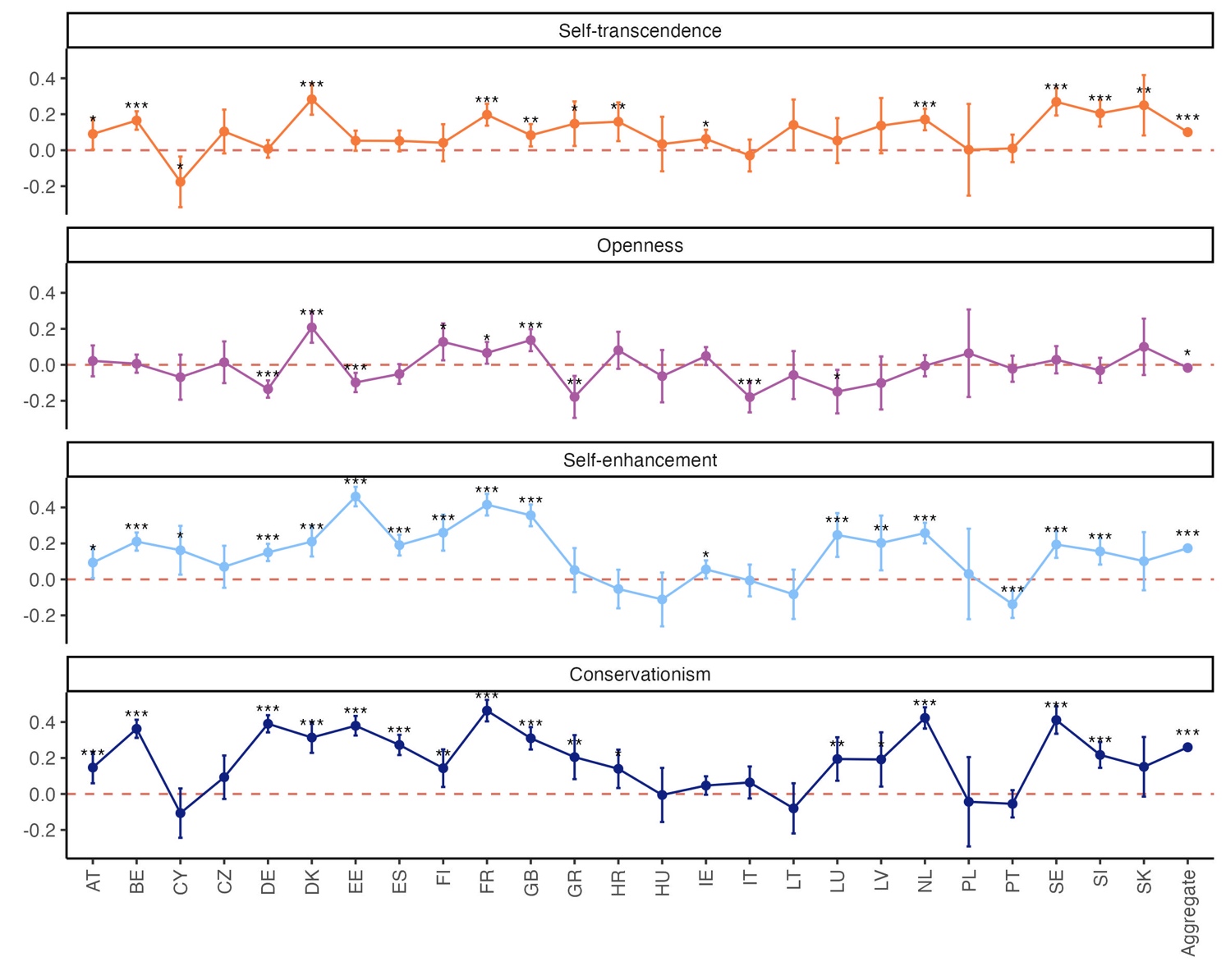


**Supplementary Figure 5.1.** Effect of being foreign-born on groups of values according to Schwartz (1992) grouped by country of residence.

Note: The countries of Romania and Bulgaria do not appear in the figure due to their lack of sufficient immigrant respondents. *** p < .001; ** p < .01; * p < .05.

## Discussion

Similarly to Supplementary Analysis 2, these results aim to clarify differential effects of being foreign-born across different groups, in this case separating the models according to the country where the data was collected and, consequently, where the respondent declares to reside. Similarly to those previous analyses, self-enhancement and conservationism display the most significant and clear effects of being foreign-born, with consistent positive associations and fewer non-significant countries that sometimes also lack enough sample for this kind of separate analysis. Self-transcendence is divided into countries where the effect is positive and others where the effect is not significant (Cyprus is the only exception, where this association is inverted and significant). Lastly, openness is the most ambivalent group of values, displaying mostly non-significant effects and both positive and negative associations for those countries that are significant.

Overall, these results are aligned with the rest of Supplementary Analyses. Furthermore, although variation can be significant across countries, it is worth noting that all models control for this variable as a random effect, thus minimising confounding effects.
